# Augmented Switching Linear Dynamical System Model for Gas Concentration Estimation with MOX Sensors in an Open Sampling System

**DOI:** 10.3390/s140712533

**Published:** 2014-07-11

**Authors:** Enrico Di Lello, Marco Trincavelli, Herman Bruyninckx, Tinne De Laet

**Affiliations:** 1. Department of Mechanical Engineering, Division PMA, KU Leuven, BE-3001 Heverlee, Belgium; E-Mail: Herman.Bruyninckx@mech.KULeuven.be; 2. Centre for Applied Autonomous Sensor Systems, Örebro University, Örebro SE-70182, Sweden; E-Mail: marco.trincavelli@oru.se; 3. Faculty of Engineering Sciences, KU Leuven, BE-3001 Heverlee, Belgium; E-Mail: Tinne.DeLaet@KULeuven.be; 4. Department of Mechanical Engineering, Section CST, Eindhoven University of Technology, 5600 MB Eindhoven, The Netherlands

**Keywords:** metal oxide semiconductor sensor, gas sensing, Bayesian inference

## Abstract

In this paper, we introduce a Bayesian time series model approach for gas concentration estimation using Metal Oxide (MOX) sensors in Open Sampling System (OSS). Our approach focuses on the compensation of the slow response of MOX sensors, while concurrently solving the problem of estimating the gas concentration in OSS. The proposed Augmented Switching Linear System model allows to include all the sources of uncertainty arising at each step of the problem in a single coherent probabilistic formulation. In particular, the problem of detecting *on-line* the current sensor dynamical regime and estimating the underlying gas concentration under environmental disturbances and noisy measurements is formulated and solved as a statistical inference problem. Our model improves, with respect to the state of the art, where system modeling approaches have been already introduced, but only provided an indirect relative measures proportional to the gas concentration and the problem of modeling uncertainty was ignored. Our approach is validated experimentally and the performances in terms of speed of and quality of the gas concentration estimation are compared with the ones obtained using a photo-ionization detector.

## 1. Introduction

Metal Oxide (MOX) gas sensors are conductometric gas sensors that have been used in a variety of applications such as medical diagnosis [1], food quality assessment [2], environmental monitoring [3] and robotics [4]. Depending on the requirements posed by the application at hand, MOX sensors have been deployed either in a Sample Flow System (SFS) or in a so called Open Sampling System (OSS). SFS is probably the most common odour handling and delivery solution. Here the gas sensors are positioned in a sensing chamber where temperature, humidity, airflow and exposure of the sensors to the target analyte are tightly controlled. SFS enables accurate gas concentration estimation and gas discrimination since it limits the effects of most of the interfering factors, but on the other hand it results in high hardware complexity, weight, and cost. On the other hand, in an OSS configuration the gas sensors are directly exposed to the external environment. This configuration is particularly advantageous in applications where payload and costs have to be minimized and especially where it is important to capture the interaction pattern between the sensors and the gas plume. Indeed, fluctuations in the sensor response are a reliable indicator of proximity of a gas source and can provide crucial information for addressing tasks like gas source localization or gas distribution mapping [5]. These fluctuations can be captured only by an OSS since a SFS would first capture the gas and then let it flow uniformly to the sensors, destroying the dynamics of the signal.

The use of an OSS entails, however, additional complications due to the slow dynamics of MOX sensors that cannot follow the fast and sudden changes in concentration due to exposure of the gas sensor to turbulent environment. In this setting the reliable estimation of the gas concentration to which the sensors are exposed is particularly difficult. In SFS the gas sensors are exposed to the gas long enough to reach a steady state that is used for the estimation of the concentration using linear or nonlinear regression models [6–9]. This strategy is unfeasible with an OSS since the sensors, due to the fluctuations induced by turbulent gas dispersal and sudden large variations in concentration, almost never reach a steady state.

To cope with all the aforementioned difficulties caused by an OSS, we propose the application of a Bayesian time-series model, named Augmented Switching Linear Dynamical System (aSLDS) [10], to MOX sensors. The proposed model, combined with a system identification procedure, allows to:

- exploit the information contained in the *transient phase* of MOX sensor, therefore not requiring the sensor to reach a steady state;
- estimate *on-line* the concentration of the gas;
- coherently *model* the uncertainties arising from the unobservable dynamics of the gas and the observed sensor noise in a *probabilistic* setting.

The proposed algorithm is tested and experimentally validated using an array of MOX sensors deployed in OSS and exposed to sudden variations in concentration of ethanol. A Photo Ionization Detector (PID) is placed in proximity of the sensors to collect ground truth measurements on the concentration to which the MOX sensors are exposed. The PID measurements are used to identify a pair of dynamical models for each MOX sensor, *i.e.*, a first-order Linear Dynamical Model (LDS) approximation is used to represent the relation between the MOX sensor output and the underlying gas concentration.

Since the MOX sensors dynamical response is different in case of an increase or decrease in gas concentration (*i.e.*, whether the sensor is in *rise* or *recovery* phase), a correct estimation of the gas concentration requires the detection of the current sensor phase.

The aSLDS deals with this problem estimating continuously the probability distribution over the sensor phases while at the same time providing an estimate of the gas concentration. This is in contrast with previous approaches that focus on change points detection in the MOX measurements [11], or system modeling approaches that focus on estimating a relative gas distribution for mapping purposes [12].

The rest of the paper is organized as follows: in Section 2 we examine the previous work in the area, focusing on both the problem of gas concentration estimation using MOX in an OSS, as well as previous applications of Bayesian time-series models. In Section 3 the experimental setup for the collection of the measurements in the proposed OSS scenario is described. In Section 4, by means of a toy example, we describe the identification of dynamical models for MOX sensors, and after the introduction of some theoretical background on Linear Dynamical Systems and Bayesian time-series model, we dwell in the details of the proposed aSLDS model. In Section 5 the experimental results are shown and discussed. Finally, in Section 6 we draw the conclusions and outline future work.

## 2. Related Works

In the following section, we introduce previous relevant work on signal processing for MOX sensors in an OSS scenario, as well as applications of the Bayesian time-series models used in this paper in different fields.

### 2.1. On MOX Sensor Modelling

In [12] the authors address the slow recovery time of MOX sensors through a system modeling approach. Different parameters for rise and recovery phases are estimated, and the phase switches are detected solely depending on the slope of the sensor measurements. In our paper, we combine probabilistically the information conveyed by the slope with the likelihood of the observed measurements conditioned on the different phase parameters. Furthermore, in addition to [12] our approach provides an absolute estimate of the gas concentration.

In [13] an array of MOX sensors is used to build an odour map with one or two sources in the wind tunnel space, and in [14] a review of research on airborne chemical sensing with mobile robots is given.

In [15] the problem of detecting phase switches in MOX sensor measurements is addressed using a constrained optimization approach. Conversely to our approach, the method requires all the measurements to be available, and therefore can only be used *offline*. Again, the problem of estimating the gas concentration in not addressed.

### 2.2. On Switching Linear Dynamical System Model

The analysis of multi-dimensional time-series is a relevant research topic in different areas such as financial forecasting, biomedical signal analysis, and computer vision. Among the most common problems in this context, we find the estimation of one or more unobservable variables from noisy measurements (which, with a slight abuse of terminology, we refer to as *inference*), and the automatic time-series segmentation and learning of segment model parameters (which we refer to as *learning*). The Bayesian framework provides a valid solution to the problem of modeling uncertainty in time-series models, and its application has a long history of success.

In Bayesian time series model, inference entails to the estimation of one more *variables* given the model parameters. In learning the estimation of the *model parameters* themselves is the goal, and their values are generally initialized from prior knowledge, then refined with a *backward* process that goes from a set observations, through the inferred variables, to the model parameters.

We will briefly explore some related previous work on inference and learning in Bayesian time-series models, limiting ourselves to approaches that rely on a probabilistic state-space formulation. A common assumption is that the time-series is generated by a Linear Dynamical System (LDS), observed via a noisy sensor. In this way, a distinction is made between the uncertainty on the dynamical model parameters (*i.e.*, the *process noise*) and noise arising from the sensor (*i.e.*, the *measurement noise*). If a single LDS is assumed to generate the measurements, then the problem of inference can be solved optimally with complexity linear in time using the Kalman Filter [16]. A more complex model, called Switching Linear Dynamical System (SLDS), assumes that there are more than one LDS describing the time series dynamics but only one of them is active at any point in time. The activation sequence of LDS is assumed to follow a Markovian dynamics but it is not observable, making the complexity of inference and learning in this model untreatable [17]. The model used in this paper names Augmented SLDS (aSLDS), and is derived from the original SLDS, adding to the model a probabilistic dependency between the estimated state and the probability of a specific LDS being active at each point in time. A more formal definition of the aSLDS will be given in Section 4. To perform inference and learning in the SLDS and aSDLS, several approximation schemes have been proposed in literature. Seminal works on inference in SLDS were developed in the fields of maneuvering target tracking [18] and econometrics [19]. These works focus on the inference problem alone, and develop approximation schemes based on pseudo-Bayesian inference. In [20] the generic inference framework of Expectation Propagation [21] is applied to perform inference in the SLDS model, while the approximate inference routine used in this paper was introduced by Barber in [10]. The proposed algorithm is based on a Gaussian Sum approximation scheme, suitable for inference in both the standard SLDS and its *Augmented* extension, named aSLDS. The SLDS and the aSLDS have been successfully applied to speech recognition [22] and automatic music transcription [23], respectively. One of the first works dealing with learning in SLDS is [24], where the authors first cast the problem in the more generic framework of Dynamic Bayesian Networks and then successfully develop a maximum likelihood learning approach to automatically segment video sequences of human walking and running. The more recent work of Fox *et al.* [25] introduces a fully Bayesian learning approach that allows to specify prior distributions on both the number of LDS and their parameters and then perform *Maximum A Posteriori* (MAP) inference from data. It has been successfully applied to computer vision, financial time-series analysis, and human motion segmentation. While the possibility of specifying prior distributions that express our prior knowledge of the physical system that we want to model is appealing, this implementation allows (until now) only to model zero-input LDS. This makes this approach not feasible in our scenario, since we need to learn a model of a MOX sensor that includes both a purely dynamic part as well as an input model.

As it will be shown, the problem of learning the SLDS model parameters has been simplified thanks to our experimental setup. Since we can control the gas input in the environment (as it will be explained in Section 3), the MOX sensors time-series is already segmented and the input is assumed to be observed using the PID measurements as ground truth. In this paper, we chose the approximation scheme proposed in [10] for *inference* in the aSLDS model. For what concerns the *learning*, we resort to a generic numerical optimization approach called Prediction Error Minimization, available as part of the MATLAB System Identification Toolbox. The off-line learned models can then be plugged into the aforementioned aSLDS inference routine, allowing to generalize our gas concentration estimation approach outside of a controlled environmental setup. The details of both learning and inference steps are detailed in Section 4.

## 3. Experimental Section

In the following section, we will discuss the characteristics of the sensors, which we are considering in this paper and present the setup of experiments used to obtain the data.

### 3.1. Metal Oxide (MOX) Gas Sensors

Metal oxide (MOX) gas sensors are, by far, the most widely used in electronic nose and mobile robotics olfaction applications. MOX gas sensors are conductometric sensors, where a change in the conductance of the oxide is measured when a gas interacts with the sensing surface. The change in conductance is approximately linearly proportional to the logarithm of the concentration of the gas over a range of concentrations [26]. There are two types of MOX sensors: *n*-type which respond to reducing gases like H_2_, CH_4_, CO, C_2_H_5_OH, or H_2_S and *p*-type which respond to oxidizing gases like O_2_, NO_2_, and Cl_2_ [27]. The response of a MOX sensor results from chemosorption and redox reactions at the surface. Since the rate of such reactions is dependent on the temperature and on the material of the surface, it is clear that the doping material of the sensing surface and the operating temperature considerably affect the sensor characteristics [26]. Typical temperatures for the sensing surface of MOX sensors lie between 300 °C and 500 °C.

**Figure 1.**
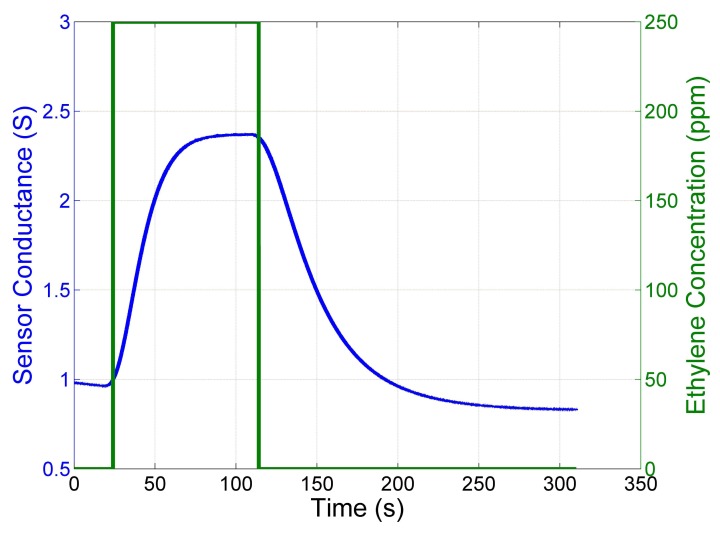
Response of a Figaro TGS2600 to a pulse of 250 ppm of Ethylene obtained in a closed sampling system.

One of the main drawbacks of MOX sensors for open sampling applications are the slow response time and the even slower recovery time. Figure 1 shows the response time and recovery time in a closed sampling system without disturbances like turbulence and advection of the airflow. The MOX sensor was exposed to a pulse of Ethylene and the sudden variation in the exposure of the sensor generates exponential-like responses. Those exponentials have different time constants depending on whether the conductance of the sensor increases (faster) or decreases (slower).

This observation forms the foundation of the proposed Switching Linear Dynamical System model.

### 3.2. Experiments and Data Collection

**Figure 2.**
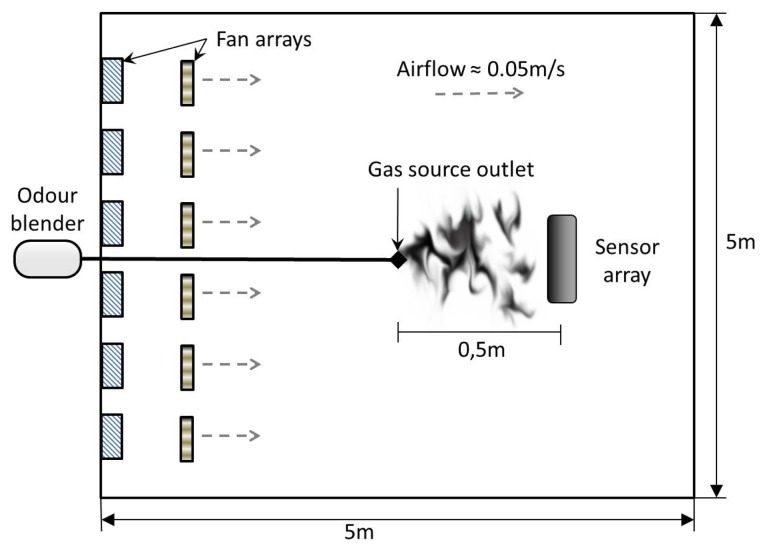
Schematics of the experimental room.

Experiments were carried out with static sensors in a 5 × 5 × 2 *m*^3^ closed room in which an artificial airflow of approximately 0.05 *m/s* was induced, see Figure 2. The airflow is created using two arrays of four fans, one placed on the floor and one on the wall. The gas source is an odor blender, a device developed by Nakamoto *et al.* [28], which allows fast switches in between different mixtures of compounds with a variable concentration. The outlet of the odor blender is placed on the floor 0.5 *m* upwind with respect to an array of eight commercial metal oxide gas sensors from Figaro Engineering [29] (TGS2600 × 2, TGS2602, TGS2611, TGS2620 ) and e2v Technologies [30] (MiCS2710, MiCS5121, MiCS5135). The selected sensors have overlapping sensitivity and they respond to a wide range of target compounds. The airflow at the outlet of the odor blender is set to 1 *L*/*min*. The sensors sampling rate is 4 Hz. A Photo-Ionization Detector (PID) sensor is also used, mounted next to the MOX array, slightly downwind.

**Figure 3.**
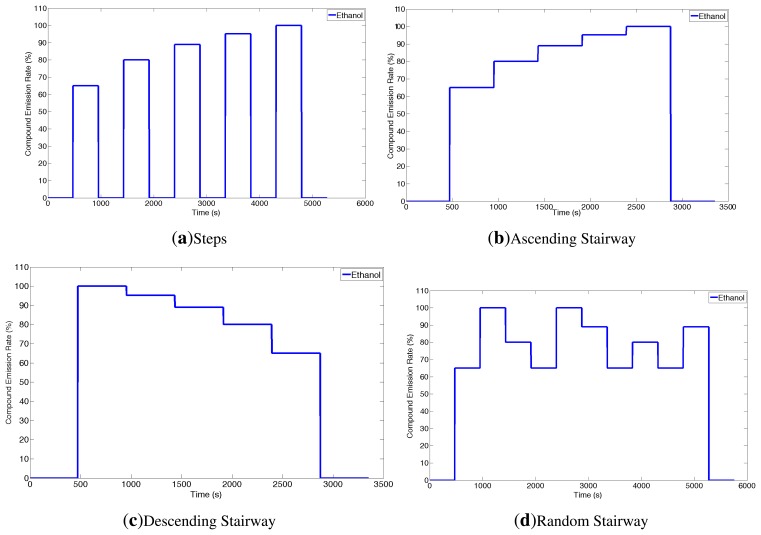
Gas source emission profiles. Profiles (**a**–**d**) are displayed. For the randomized profile (**d**), one exemplary instance is displayed.

The analyte selected for these experiments is ethanol. In order to create a database that allows to study the dynamic behavior of the sensors when consecutively exposed to different analytes, four different odor emitting profiles have been applied. For all these profiles, the gas source emits clean air for two minutes and the signal of sensors during this period is assumed as a baseline. At the end of all the experiments, the source emits again clean air for 2 min. Figure 3 shows the intensity profile for the gas source in the various emitting profiles. A total of 54 experimental runs have been performed. The concentration of analyte released varies from 35 to 200 ppm.

The control signal of the odor blender is used as ground truth for the change point time and provides the time at which the source changes the emission modality. However, in order to know the change point time at the sensors' location, we need to estimate the time it takes the gas to travel from the gas source to the sensor location. Since the sensors are placed 0.5 *m* away from the location of the source outlet and a steady air flow of 0.05 *m*/*s* is induced, the delay time between change times at source and sensor location is estimated to be 10 *s*. The estimation of the delay has been validated with a cross correlation analysis between the control signal of the odor blender and the signal of the MOX sensors in the Steps experiments (see Figure 3a).

## 4. Estimating Gas Concentration with MOX Sensors and the (Augmented) Switching Linear Dynamical System Model

As shown in Figure 1, the time response of a MOX sensor to a step of gas concentration evolves approximately according to two exponential functions with different time-constants, depending on whether the concentration of the gas is increasing (which can be defines as *rise* phase) or decreasing (*recovery* phase). Since the time-response of a first order Linear Dynamical System is indeed an exponential function that depends on the system time constant, the input model and time, it seems a natural choice to model a MOX sensor using two LDS, one for each of two phases. Given the LDS parameters, it is possible to use the aSLDS to compute on-line the activation probability of the two models, and then estimate the underlying gas concentration, while taking into account the disturbances that affect the signal in an OSS.

Our approach can be decomposed in two separate steps:

- **Off-line**: *learning* the LDS parameters for each phase and each sensors in the array.
- **On-line**: use the learned parameters to *infer* the current sensor phase and the gas concentration.

A generic LDS formulation describes by means of differential (or difference, in case of discrete-time formulation) equations, the free-evolution of the *state* of a system, its dynamic response to an external *input*, and how the state is related to the *measurements*.

Thanks to our experimental setup, we can learn the LDS parameters for each sensor and phase separately, using standards numerical methods developed in the field of System Identification. Once the LDS parameters are known, the problems of detecting the current sensor phase and estimating the gas concentration can be solved concurrently via *inference* in a aSLDS model. This probabilistic approach allows to model and take into account the sources of uncertainties arising from modeling errors and the aforementioned difficulties arising in OSS (namely, the turbulences in the environment that prevent the MOX sensors to reach a steady state), and to provide confidence measures for each of estimated variables.

In Section 4.1 we introduce a toy example suitable to explain the identification of the dynamical parameters of a MOX sensor. We will progressively increase the complexity of the proposed sensor model in Section 4.2, as well as provide the theoretical background for understanding the Switching Linear Dynamical System model and its Augmented extension.

Finally, in Section 4.3, the results obtained applying the aSLDS to the toy examples are shown and explained.

### 4.1. Identification of Dynamical Parameters: A Toy Example

In this section, we will the describe how the time response of a MOX sensor can be described using two LDS and how their parameters can be learned from data. For each of the two phases *p* (*rise* and *recovery*), each MOX sensor s in our array is modeled as a discrete-time first order linear dynamical system (LDS), defined as follows:

(1) $$\begin{array}{ll} {\text{x}}_{t+1} & =A_{sp}{\text{x}}_{t}+B_{sp}{\text{u}}_{\text{t}}\\ {\text{y}}_{\text{t}} & =C_{sp}{\text{x}}_{\text{t}} \end{array}$$

Each sensor is treated independently, and the same input vector **u_t_** is assumed for all sensor of the array. The notation is defined as follows (from now on, we will drop the indices *s* and *p* where possible for readability):

- x*_t_*_+1_ is the (unobservable) state vector of the sensor at time *t* + 1, and **A** is the *dynamics matrix.* During the the system identification step, the vector has only one component, the (unobservable) true sensor conductance at time *t*, indicated in the by *x_t_.* The state vector will be extended later on, for the on-line gas concentration estimation.
- *B* is the *input matrix* and **u_t_** is the *input* at time *t*, *i.e.*, the gas concentration, which is assumed to be available at this stage;
- *C* is the *sensor matrix* and **y_t_** is the *observation* vector at time *t*, which in our case is simply the measured MOX sensor conductance at time *t.*

The parameters *A, B*, and *C* are assumed to be time-independent. Since the chosen model is a first-order, fully observable LDS, all the parameters are scalars. In particular, for each phase *p* and sensor *s*:

(2) $$\begin{array}{ccc} A_{sp}=\tau_{sp}, & B_{sp}=b_{sp}, & C_{sp}=1 \end{array}$$

where *τ_sp_* is related to the *time-constant* of the system, and by setting *C* to 1 we are assuming that no scaling is involved between the true sensor conductance (*i.e.*, the *state*) and the observed one (the *measurement*)*.*

**Figure 4.**
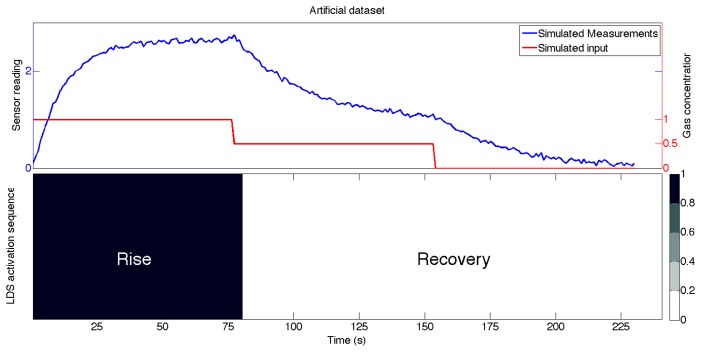
Data for toy example. (**Top**) The simulated sensor measured and the input time series are shown. The time constants for the rise and recovery phases are different. (**Bottom**) the activation sequence of the *rise* and *recovery* LDS models.

As a toy example to explain the system identification and later the aSLDS inference, an artificial dataset was generated as follows. First, two first-order LDS as in Equation (1) with different values of *τ* and *b* were defined. We then simulated the time-response of the sensor model to a sequence of step inputs of variable amplitude. The *rise* time-constant is used when the input increases with respect to the previous value, while the *recovery* model is used in the other case otherwise. Gaussian noise is added to the state to simulate measurement noise. We define *rise* and *recovery* phases according to the input, not the sensor output. We consider the sensor to be in *rise* phase for as long as the input at time *t* is not smaller than the one at time *t* – 1, and in *recovery* phase as long as the input at time t is not bigger than the previous one. This is because the two LDS are distinguishable only from their transient response, so for our application there is no advantage in specifically modeling the steady-state phases. The generated dataset is shown in Figure 4.

Assuming that the input **u_t_** is known, the LDS parameters *τ* and *b* can be estimated from data using numerical optimization methods. This assumption is automatically met in this toy example, but it can also be met using our experimental setup, where we can control the gas emission in the environment and accurately measure the gas concentration the MOX sensors are exposed to using the PID sensor. It should be noted that the PID sensor, as well as a ground truth gas concentration measure are necessary only during this learning phase, which can be interpreted as a sensor calibration. Later on, we will show that plugging the learned sensor parameters in the aSLDS models allow to obtain a gas concentration estimate comparable to the one obtained with the PID using only the MOX sensors.

In this paper, we used the MATLAB pem function for the identification of the dynamical parameter of each MOX sensor. The acronym stands for *Prediction Error Minimization* and the function is part of the System Identification Toolbox. This method falls in the category of so-called *grey-box* methods, which allows to specify initial guesses for both the model parametric form and the parameter values. Given these initial guesses, and a set of experiments consisting of the sensor measurements y_1:T_ and the input u_1:T_, the pem function tries to minimize the weighted norm of the prediction error, *i.e.*, the difference between the measured output and the predicted output of the model.

A more detailed analysis of the identification method or a comparison between different methods is beyond the scope of this paper. To give a quantitative evaluation of the accuracy of the estimated parameters, for the toy example shown in Figure 4 we simulated a zero-mean Gaussian measurement noise with covariance equal to 10^−3^ . The error on the estimated dynamical parameters for both phases is approximately 5%, as well as the overall prediction error.

### 4.2. Inference in the aSLDS Model: Input and Active Phase Estimation

During the offline learning step explained in the previous section, the LDS parameters for each sensor and each phase are identified from the sensor measurements, assuming that the input sequence (*i.e.*, the ground truth gas concentration) is known. In our application we reverse the problem: we want to *estimate* the gas concentration using the sensor measurements and the knowledge of the sensor LDS parameters. To achieve this goal, it is necessary to move the input u_t_ into the state vector of the LDS, and consequently to specify a dynamical model for the input.

The dynamics of the input, (*i.e.*, the gas concentration) is not known *a-priori*, and it would be pointless to learn it from data, since learned dynamics will fit the set of experiments used for learning, but will likely not generalize to different experiments. For this reason, we make the assumption that the input dynamics is constant, but perturbed by Gaussian noise at each point in time. This idea is derived from a common approach in target tracking applications. In this domain, it is common to assume that the target moves according to a n-order *constant derivative model* (e.g., constant velocity (CV) or constant acceleration (CA)). Placing a Gaussian noise dynamics on the n+1 order derivative (e.g., a random acceleration in a CV model), allows to capture the target trajectory variability, compensating for the restraining assumptions made by the dynamical model [18]. In the target tracking domain, it is also common to assume multiple dynamical models (e.g., a target performing a *left turn* or a *right turn)*, and to use the Switching Linear Dynamical System model to detect a target maneuver and select the *best fitting* dynamical model to estimate the target state (usually its position and velocity). Similarly, we will adopt the SLDS model to detect what is the most likely current phase of the sensor, and estimate accordingly the underlying gas concentration. Furthermore, in an attempt to include in our model as much knowledge as possible about the dynamics of the process, we would like to incorporate the notion that a phase switch in the MOX sensor can be detected observing the trend of the measurements, (*i.e.*, if the trend of the measurements is the negative, a switch from *rise* to *recovery* is more likely, while the opposite is true for a positive trend).

#### 4.2.1. The Extended LDS Formulation

The fact that the trend can be used as an indicator for a phase switch justifies the adoption of the aSLDS model, where the dependence between the current estimate of the state and the probability of a specific LDS being active is explicitly modeled. Since we cannot directly measure the trend, we resort to a numerical approach. We approximate the trend *ẋ* using the backward difference of measurements divided by the sensor sampling time Δ*t*.

(3) $${\dot{x}}_{t+1}=\frac{x_{t+1}-x_{t}}{\Delta t}$$

To reduce the sensitivity to measurement noise, we low-pass filter the estimated trend using a smoothing coefficient *α* to obtain a smoothed approximate derivative *ẋ^s^*.

(4) $$\begin{array}{cc} {\dot{x}}_{t+1}^{s}=\alpha {\dot{x}}_{t+1}+(1-\alpha ){\dot{x}}_{t}^{s} & 0<\alpha <1 \end{array}$$

The newly extended LDS formulation for each sensor *s* and each phase *p* is now as follows:

(5) $$\begin{array}{cccc} {\text{x}}_{\text{t}}=\left[\begin{array}{c} x_{t}\\ u_{t}\\ {\dot{x}}_{t}^{s} \end{array}\right], & A_{sp}=\left[\begin{array}{ccc} \tau_{sp} & b_{sp} & 0\\ 0 & 1 & 0\\ \frac{\alpha (\tau_{sp}-1)}{\Delta t} & \frac{\alpha (b_{sp})}{\Delta t} & \left(1-\alpha \right) \end{array}\right], & B_{sp}=0, & C_{sp}=\left[\begin{array}{ccc} 1 & 0 & 0 \end{array}\right] \end{array}$$

where:

- the state vector x_t_ is extended to include the unobservable input *u_t_* and the smoothed derivative ${\dot{x}}_{t}^{s}$, as well as the sensor conductance *x_t_*.
- the dynamical matrix *A_sp_* now describes the evolution of the extended state. The first row of the matrix ties together the sensor conductance *x_t_* and the unobservable input *u_t_*, via the input scaling factor *b_sp_*. The second row describes the input dynamics, assumed to be constant. While this assumption might look restrictive, it will be shown later that the additive process noise and the linear relationship with the estimated sensor state will allow to capture the input variability.
  The last row computes the smoothed derivative of the sensor state, using the smoothing factor *α*, and assuming that the sensor sampling time is Δ*t*.
- The *B* matrix is now zero, since the input is no longer observable.
- the sensor matrix *C* contains 1 in correspondence of the first element of the state vector x_t_ to model the fact that we can observe noisy realizations of the the sensor conductance *x_t_*, while the zeros model the fact that the input *u_t_* and the estimated trend ${\dot{x}}_{\text{t}}^{\text{s}}$ cannot be observed.

#### 4.2.2. Inference in LDS: Kalman Filter formulation

The Kalman Filter (KF) [16] is the optimal choice to estimate on-line the state of an LDS from noisy measurements. In our application, however, it is not sufficient to use a KF, because the problem of estimating the current sensor phase must also be solved at the same time. Nevertheless, the aSLDS inference algorithm utilizes a KF as a subroutine, as it will be shown later. While a detailed analysis of the Kalman Filter algorithm and properties are beyond the scope of this paper, we will shortly describe here the logic of a KF and mention two crucial parameters introduces by this estimation algorithm, namely the *process* and the *measurement noises.*

In short, the Kalman Filter is an iterative algorithm consisting of two phases:

1. **prediction**: where the current estimate of the state is propagated one step forward in time using the LDS dynamical matrix *A*, and an expected measurement is computed via the observation model;
2. **correction**: where the predicted measurement is compared with the most recent one, and the estimate of the state is updated *optimally* taking into account the uncertainty on the dynamical model and the sensor noise;

The word *optimally* is to be interpreted in a statistical sense: the uncertainty on the dynamical model, modeled via the *process noise* and the one on the sensor, modeled via the *measurement noise*, are weighted to minimize the covariance of the error on the estimated state (more formally, the KF is the *minimum variance unbiased estimator* (MVUE) of the LDS state).

The role of the process noise is to compensate for the limiting assumptions made by the dynamical model or for the lack of accuracy of its parameters. In the case of our LDS, the dynamical model assumes the input to be constant, which is a valid approximation for small sampling times. However, since we know that the gas concentration in an OSS will not be constant over the duration of a whole experiment, we need to allow the input estimate to be “perturbed” and then optimally refined on-line as new measurements are available.

The process noise represents how much we expect the state vector at time *t* + 1 to deviate from the estimated value obtained propagating the state vector at time *t* using the dynamics matrix. This deviation can be due to the error in the estimation of the dynamical parameters, as well as due to a change in the gas concentration between *t* and *t* + 1. Similarly, the measurement noise model represents how much the sensor reading at time *t* value deviates from the true conductance of the sensor.

More in details, the process and measurement noise are incorporated in our LDS formulation as follows:

(6) $$\begin{array}{ccc} {\text{x}}_{t+1} & = & A{\text{x}}_{t}+\eta_{k}^{x} \end{array}$$

(7) $$\begin{array}{ccc} {\text{y}}_{t} & = & C{\text{x}}_{t}+\eta_{k}^{y} \end{array}$$

(8) $$\begin{array}{ccc} \eta_{k}^{x}∼N\text{(}\bar{x},\text{Q)} & , & \eta_{k}^{y}∼N\text{(}\bar{y},\text{R)} \end{array}$$

where:

- *A* and *C* are the dynamics and observation matrix, respectively, introduced in the previous section;
- $\eta_{k}^{x}$ is the additive *process noise* and $\eta_{k}^{y}$ is the additive measurement noise;
- *x̄_k_, Q*, and *ȳ_k_*, *R* are the mean and the covariance of the additive process and measurement noise, respectively Both are assumed to be Gaussian;

The *Q* and *R* matrices, and in particular their ratio, have a crucial impact on the optimal estimation of the LDS state. It must be noted that the state components *x_t_* and *u_t_* (*i.e.*, the estimated sensor state and input at time *t*) are coupled via the dynamical matrix *A.* For this reason, while in our model the process noise covariance can be specified using a single scalar *q*, acting as an external perturbation on the input, the process noise covariance matrix *Q* must take into account this coupling (*i.e.*, how the noise on the input *u_t_* is propagated to the state *x_t_*)*.*

Process noise covariance matrices for n-order constant derivative dynamical models are commonly derived in literature [18], but those derivations do not apply directly to our application. Given our LDS formulation, the computation of the process noise matrix requires the solution of the following integral:

(9) $$Q=\int_{0}^{T}e^{A\tau }GqG^{T}e^{A^{T}\tau }d\tau$$

where *q* is process noise covariance (in our case a scalar), *A* is the dynamic model matrix and *G* is the error input matrix, specifying how the input affects the dynamics of the state. In our case, *G* = [0 1], meaning that the process noise affects directly only the second state variable (*i.e.*, the input *u_t_*)*.*

The integral in Equation (9) is computed numerically using the matrix exponential approximations in [31]. The measurement covariance, conversely, is in our case simply determined by a single scalar value r.

#### 4.2.3. The aSLDS Model

If we could approximate the MOX sensor time response with a single LDS, the Kalman Filter would be the optimal estimator for the state variables.

Since we are dealing with two different LDS for each sensor *s*, and the sensor phase *p* is not directly observable, we need to look beyond the simple KF and extend the model to take into account the unobservable *switches* of the sensor phase. This leads to the use of the aSLDS model, and this choice is justified by two fundamental features:

1. the aSLDS explicitly models the presence of multiple LDSs, but rather than performing a hard assignment, (*i.e.*, allowing only one of the system to be active at each point in time), it defines a *probability distribution* over the possible LDS activation. This feature is particularly helpful in making the estimation process robust to noisy measurements and modeling errors.
2. in the aSLDS, the dependence between the current estimate of the state and the probability of a phase switch is explicitly modeled. This feature allows to use the estimated trend *ẋ^s^* as a “hint” for a phase switch.

**Figure 5.**
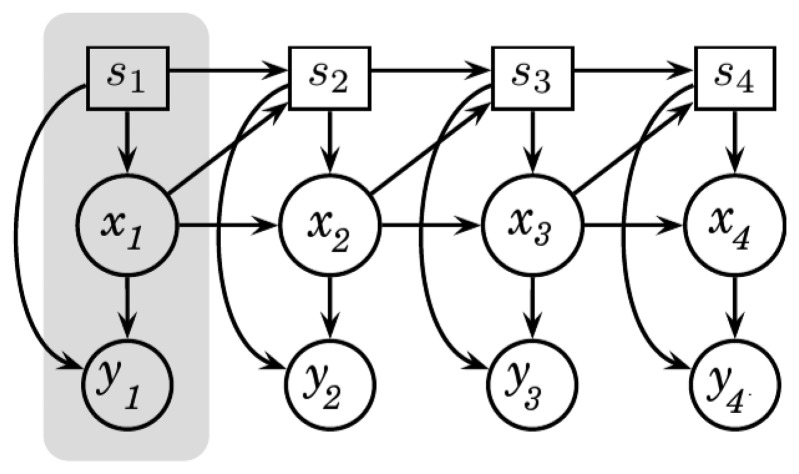
Bayesian Network representation of the Augmented Switching Linear Dynamical System. A slice is highlighted in gray.

To better understand the aSLDS model, it is worth familiarizing with its Bayesian network representation, presented in Figure 5.

A Bayesian Network is a probabilistic graphical model that represents, by means of a *directed acyclic graph*, how a joint probability distribution can be factorized in the product of a set of conditional distributions. The factorization induced by the graph allows the development of inference algorithm that are specific for some standard graph topologies aiming at lowering the complexity of otherwise intractable inference problems. Bayesian Network models for time-series exhibit peculiar topological features, and are referred to as Dynamic Bayesian Networks (DBNs) . For a deeper understanding of the most common DBN models and their inference algorithms we refer the reader to [32,33]. Each node of a DBN represents a random variable (r.v.) and, for each edge, a conditional probability distribution must be defined between the *parent* node (*i.e.*, the one containing the tail of the arrow) and the *children* node (*i.e.*, the one pointed by the arrow the head). A DBN is modular graph, where each module is called slice, consisting in the set of nodes indexed with a specific time index *t*.

The aSLDS model defines, for each slice/time *t*, three different r. *vs.* (we modify the notation introduced in [10], to be consistent with the KF notation previously introduced ) :

- the *switch variable s* ∈ 1, …, *S*, a categorical r.v. representing the probability of each of the possible LDS being active at time *t* . In our scenario, *s* corresponds to the sensor phase *p*, and is therefore a binary r.v. (*S* = 2) , indicating the probability that the sensor is in *rise* or *recovery* phase.
- the *hidden variable x* ∈ *R^N^*, the continuous r.v. representing the state vector of each LDS. *N* is the size of the state-space, in our case *N* = 3.
- the *visible variable y* ∈ *R^M^*, a continuous or discrete r.v. representing the observation vector. In our case, *y* is continuous and *M* = 1.

Using the rules for conditional independence in Bayesian Networks [32], the joint probability of the variables in the aSLDS model can be factorized as follows:

(10) $$p(y_{1:T},x_{1:T},s_{1:T})=p(y_{1}|x_{1},s_{1})p(x_{1}|s_{1})p(s_{1})\prod_{t=2}^{T}p(y_{t}|x_{t},s_{t})p(x_{t}|x_{t-1},s_{t})p(s_{t}|x_{t-1},s_{t-1})$$

The conditional probability distributions on the *r.h.s.* of Equation (10) are defined as follows:

- *p*(*s*_1_) is the prior probability of the switch variable. In our application, both phases are assumed equally likely at the beginning of the inference (*i.e.*, *p*(*s*_1_ = *rise*) = *p*(*s*_1_ = *recovery)* = 0.5).
- *p*(*x*_1_|*s*_1_) is the prior probability of the state given the initial switch state. It is a conditional Gaussian distribution, and its only role is to initialize the inference routine.It requires the definition of two additional parameters, namely the prior mean *x̄*(*s*_1_) and the prior covariance *P*(*s*_1_):
  (11) $$p(x_{1}|s_{1})=N(\bar{x}(s_{1}),P(s_{1}))$$
  In theory, the initial state can be dependent on *s*_1_. The practical choice of using a vague prior independent of *s*_1_ is made in our application.
- *p*(*y_t_*|*x_t_, s_t_*) is the conditional distribution of the observed variable at time *t* conditioned on the state and the switch variables, also at time *t.* The dependency of the variable *y* on *s* expresses the fact the observation model parameters could in theory be different among systems, while in our application they are the same for both *rise* and *recovery* phases. Since observed variable y is linearly related to the the state variable x , the resulting conditional distribution is also Gaussian:
  (12) $$p(y_{t}|x_{t},s_{t})=N(\bar{y}(s_{t})+C(s_{t})x_{t},R(s_{t}))$$
- *p*(*x_t_*|*x_t_*_−1_*, s_t_*) is the conditional distribution of the state variable at time *t* conditioned on the previous state and the current switch variable. The switch variable *s* acts as a selector of the LDS to be used to propagate the state forward in time, and the resulting distribution is again Gaussian:
  (13) $$p(x_{t}|x_{t-1},s_{t})=N(\bar{x}(s_{t})+A(s_{t})x_{t-1},Q(s_{t}))$$
  where the process noise parameters and the dynamical model are depending on the value of the switch variable. In our application, *x̄* is a zero vector, while the dynamic and covariance matrices *A* and *Q* are different for the rise and recovery phases.
- *p*(*s_t_*|*s_t_*_−1_*, x_t_*_−1_) is the conditional distribution of the switch variable *s_t_* conditioned on its previous value and on the previous estimate of the state variable. In the simple SLDS model, the dependence on the state variable is dropped, and a Markovian transition model is used. In our application, we exploit the dependence on the state to bias the model towards a transition depending on the value of the estimated trend ${\dot{x}}_{t-1}^{s}$. More in detail, we start from the classical Markovian model for the probability of a switch:
  (14) $$p({\text{s}}_{t}|s_{t-1})=\{\begin{array}{ll} p & \text{if}\;s_{t}=s_{t-1}\\ 1-p & \text{otherwise} \end{array}$$
  where *p* is the self-transition probability, for which a high value (e.g., 0.9) is a typical choice to penalize unrealistically fast dynamic switches. We then extend this model introducing the dependence on ${\dot{x}}_{t-1}^{s}$ as explained in Table 1:

The bias term *b* is introduced to favor a phase switch depending on the previous state and the estimated trend. Rather than looking only at the sign of *ẋ^s^*, the value of *b* is made dependent on its absolute value.

**Figure 6.**
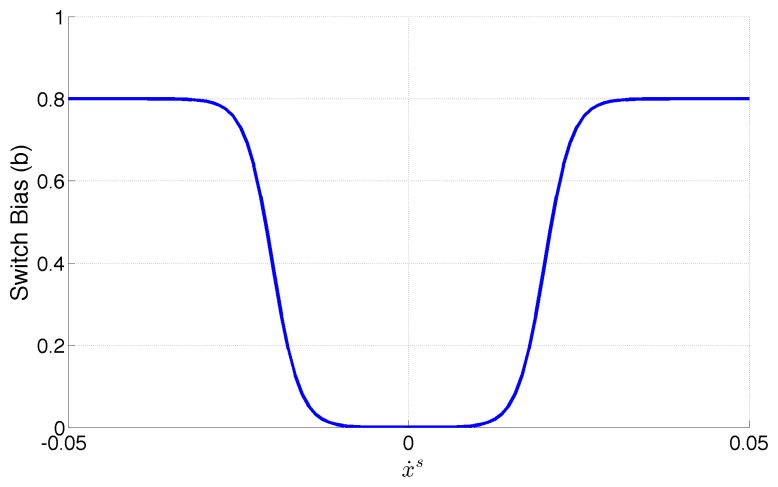
The aSLDS transition model.

The resulting model, shown in Figure 6 is obtained as a combination of two sigmoid functions, symmetric with respect to 0. The “hole” shaped area around 0 determines the range of values of the slope that have little effect on the bias. Tuning the sigmoid parameters influences the area of the “hole”, as well as its slope, making the bias model more or less reactive to the slope (and therefore sensitive to the noise on its estimate).

Exact inference in the aSLDS model is untractable, because its complexity scales exponentially with time [17]. To deal with this problem, several approximation scheme have been proposed in literature. We adopted the one of Barber [10], named *Expectation Correction*, whose *MATLAB* implementation is available in the *BRML toolbox* [34]. The method is numerically stable and compares favorably against alternative approximation schemes in terms of both speed and accuracy.

### 4.3. Results on Toy Example

Given a set of measurement *y*_1:_*_T_*, the *Expectation Correction* (EC) approximation scheme for the inference on the aSLDS computes the most likely switch sequence *s*_1:_*_T_*, and estimates the state *x*_1:_*_T_* conditioned on the switch probability distribution.

**Figure 7.**
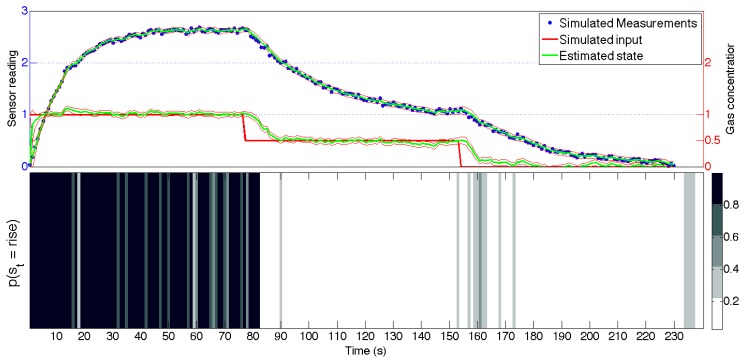
The aSLDS inference results on the artificial dataset. (**Top**) Measurements and estimated input (**Bottom**) Grayscale representation of *p*(*s_t_*) = *rise*, where black is 1 and white is 0.

Figure 7 shows the results on the artificial data explained in Section 4.1, and the estimated state is plotted on top of measurements. It must be noted that during the inference, the input variable is not observed, but obtained from the noisy measurements after estimating the switch variable distribution. It can be seen that the amplitude of the input steps is correctly estimated, while a delay is introduced around the input discontinuities. This is due to the constant dynamics assumed for the input in the dynamic matrix A in Equation (5). Nevertheless, the aSLDS is able to compensate for the violation of this modeling assumptions and to recover the correct value after a few measurements. At each point in time, the aSLDS computes the likelihood of a possible switch. By propagating multiple transition hypotheses as well as estimating the probability distribution over *s_t_*, the model is able to detect switches in the dynamical model, and estimate the input accordingly.

At the bottom of Figure 7, the probability distribution of the switch variable is represented in grayscale, for visual comparison with the ground truth shown in Figure 4. From this part of the figure, it can be noted that the the probability of a sensor phase is high (or low) where the signal is rapidly changing like, for example between 0 and 15 s or around 85 s. Conversely, when the system is in steady-state, many gray bands can be seen, because the measurements themselves contain little information, but the transition model is biased toward keeping the previous values. The delay introduced by the filter is related to the bandwidth of the filter, which is related to ratio of the process and measurement noise amplitude *q* and *r*. The amplitude of *q* in Equation (9) will also affect the covariance of the estimated state, represented in the figure by the 2 − *σ* confidence interval plotted around the estimated state variables. The tuning of *q* and *r* is a crucial step in the application of the aSLDS model. Guidelines on settings these parameters rely on statistical tests, and can be found in [35].

Finally, it can be noted how the input estimate reaches a steady state faster than the measurements. This proves the validity of the aSLDS model for gas estimation in our OSS scenario, where the MOX sensor often do not reach a steady state. Using the aSLDS, the gas concentration can be estimated much faster, exploiting the information contained in the sensor transient response.

## 5. Results

In Section 4, we have shown how the aSLDS allows, given a time-series, to estimate the probability distribution of a set of possible dynamical models being active at each point in time as well as the corresponding unobservable input sequence. In this section, we present the results of the aSLDS approach to gas concentration estimation using MOX sensors. In our application, the dynamical models are given by the 8 MOX sensors, each one exhibiting different dynamical properties for each phase, and the unobserved input to be estimated is the underlying gas concentration. First, we use the data collected from a subset of the experiments described in Section 4 to learn the dynamical parameters of our sensor array, using the PID measurement and the known gas emission sequence of the odor blender as *ground truth* for the real gas concentration and the sensor current phase, respectively. Then, we apply the aSLDS to estimate *on-line* the gas concentration using only the MOX sensors measurements for the set of experiment not used during learning. The algorithmic complexity of the our approach is O(512) for each sample of each sensor. In our MATLAB implementation (running on a standard laptop), it takes 539 s. to process an experiment with a duration of 1437 s. Therefore an on-board, real-time implementation is feasible.

We measure the performance of our approach comparing the estimated gas concentration to the ground truth PID measurements.

### 5.1. Pre-Processing and Learning

In order to learn the dynamical parameters of the MOX sensors, a set of pre-processing steps are required. We select one trial from three of the gas emission profiles shown in Section 3, *i.e.*, the datasets *Steps, Ascending Stairway*, and *Descending Stairway.* In this way, we make sure that both phases of the sensor are observed, for different values of the gas concentration. The MOX measurements are then segmented according to the odor blender input signal in *rise* and *recovery* phase, labeling the time instants when the gas concentration increases or decreases with respect to the previous values. In this way, we can separate the data to be used for learning the models of each phase. For the purpose of drift compensation and dynamic range enhancement, the raw sensor readings are divided by the sensor baseline identified by the measurement obtained before emitting gas in the environment, as suggested in [12]. The measurements are then inverted, *i.e.*, transforming the sensor resistance in conductance values. For the range of gas concentration used in our experiments, we found that the best model to describe the relationship between the MOX conductance and the gas concentration measured in ppm with the PID is a log-linear one. Therefore we compute the logarithm of the measurements, and after finding the maximum values over all the experiments for each sensor, we scale the resulting values between 0 and 10.

**Figure 8.**
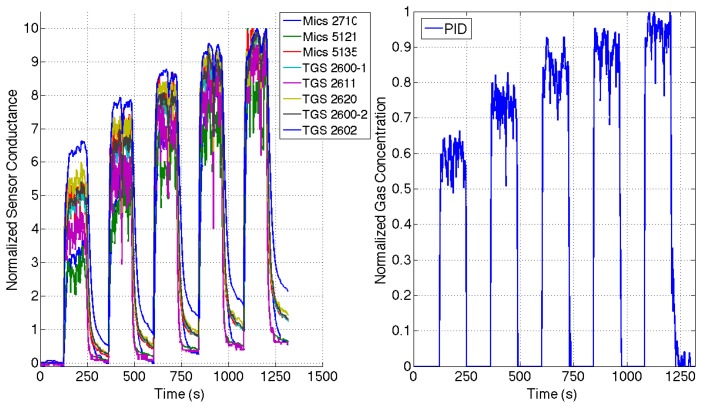
Pre-processed MOX and PID data for one trial of the *Steps* dataset.

The PID measurements are pre-processed in a similar way: we remove the baseline, compute the logarithm of the concentrations and we scale them between 0 and 1. This is done to keep the MOX measurement and the PID ones in a range that makes the system identification phase numerically more stable and robust to noise. Figure 8 shows an example of the resulting pre-processed MOX and PID measurements for a one trial of the *Steps* dataset.

None of the pre-processing steps compromises the *on-line* nature of our approach. The scaling coefficients can be computed beforehand, and nothing prevents the aSLDS to estimate a gas concentration bigger than the ones observed during learning.

After the pre-processing, we apply the pem method introduced in Section 4 to identify the dynamical parameters for each sensor and phase using the data from the selected experiments.

### 5.2. Sensor Phase and Gas Concentration Estimation

Once the dynamical parameters of each phase for each sensor are identified, we plug them in the aSLDS inference routine and estimate the gas concentration for the set of experiments not used in the learning phase. In particular, we are left with eight experiments, two for each of the datasets *Steps, Ascending Stairway, Descending Stairway* and *Random Stairway*. The values of the process and measurement noise amplitude *q* and *r* were determined experimentally, following the guidelines contained in [35]. The choice of finding values acceptable for all the sensor models is made for simplicity, but in general these parameters can be fine-tuned to each sensor. The smoothing parameter *α* for the estimated trend *ẋ^s^* is set to 0.1, resulting in a low-pass filter with cutoff frequency at approximately 0.5 Hz.

**Figure 9.**
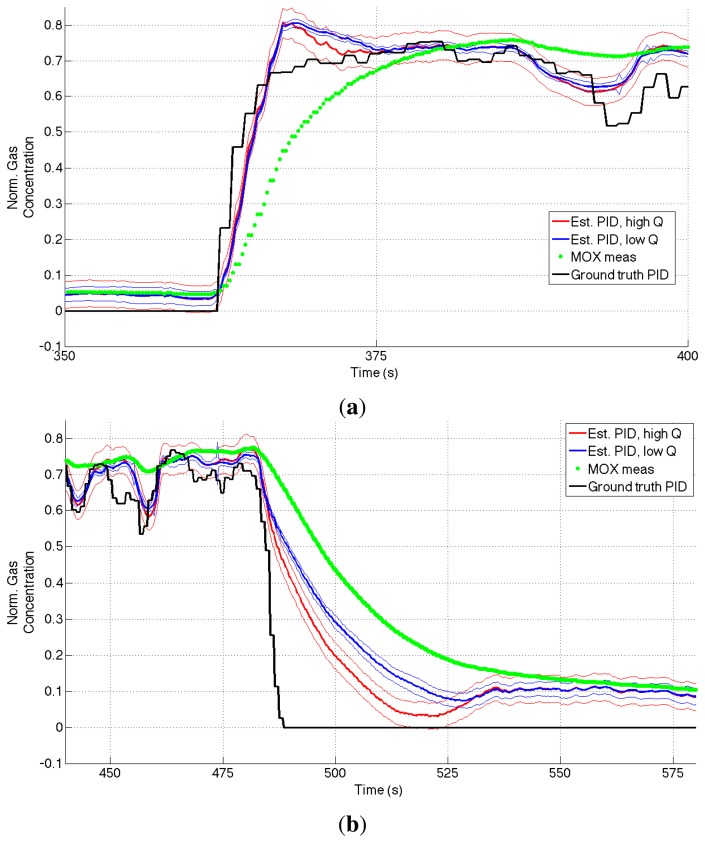
Gas concentration estimation results for *rise* (**a**), and *recovery* (**b**) phases of a *Steps* dataset using different values for the process noise amplitude *q* . For each color, the thicker line indicates the mean value, the thinner ones the 2 − *σ* interval.

Figure 9 shows the difference between the simple scaled MOX measurements and the gas concentration estimate obtained using the aSLDS model for a portion of the step dataset, using two different values for the parameter *q.* It can be noted how the aSLDS estimated input follows the PID ground truth much more closely, especially where the gas concentration exhibits sudden changes.

As explained in Section 4 we modeled the hidden input dynamic as wise constant. The process noise parameter *q* represents our confidence in the dynamical model. Therefore, increasing *q*, allows the aSLDS to violate the constant assumption for the input, resulting in a faster input estimate in reaction to a gas concentration step. The price to pay for this increased responsiveness is in terms of the increased covariance of the estimate, shown in the figure using the 2 − *σ* confidence interval.

**Figure 10.**
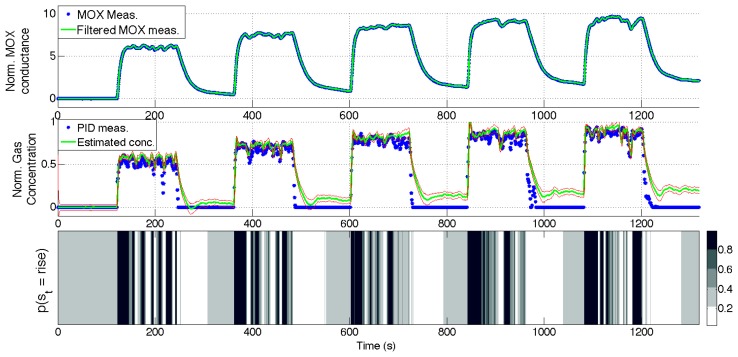
aSLDS inference results for an experiment of dataset *Steps*. (**Top**) MOX measurements, (**Middle**) Gas concentration, (**Bottom**) Sensor phase probability distribution.

**Figure 11.**
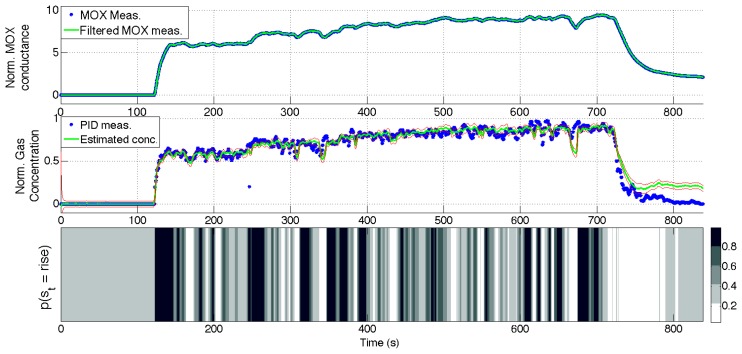
aSLDS inference results for an experiment of dataset *Ascending Stairway*. (**Top**) MOX measurements, (**Middle**) Gas concentration, (**Bottom**) Sensor phase probability distribution.

**Figure 12.**
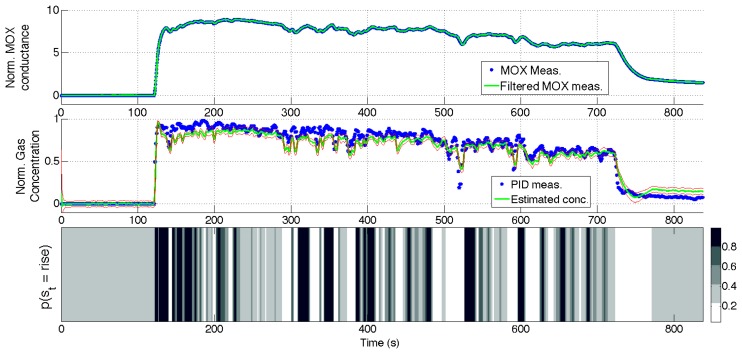
aSLDS inference results for an experiment of dataset *Descending Stairway*. (**Top**) MOX measurements, (**Middle**) Gas concentration, (**Bottom**) Sensor phase probability distribution.

**Figure 13.**
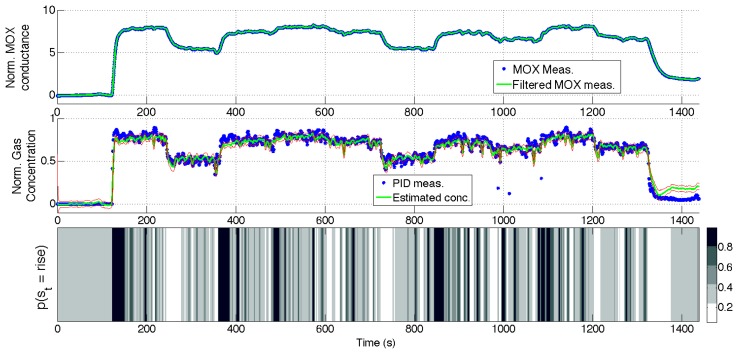
aSLDS inference results for an experiment of dataset *Random Stairway*. (**Top**) MOX measurements, (**Middle**) Gas concentration, (**Bottom**) Sensor phase probability distribution.

Figures 10, 11, 12 and 13 show the full state estimated using aSLDS inference for four experiments not used in the learning phase, one for each dataset, for one of the sensors. The filtered MOX measurements are plotted together with the raw ones, and the estimated gas concentration is plotted on top of the PID measurements. It is useful to remind the reader that the PID measurements are *not used* in this phase, as the gas concentration is estimated only from the MOX measurements and the dynamical models previously learned. The estimated current sensor phase distribution, is also shown at the bottom of each figure. The dataset *Steps* is the most useful to highlight the features of the aSLDS gas concentration estimation. The switch probability graph, at the bottom of Figure 10, represents the probability of the sensor being in *rise* phase using a grayscale colormap, where black is 1 and white is 0. It can be noted how the phases are correctly estimated in correspondence of the transient response of the MOX sensor to gas concentration steps, clearly identifiable from the deep black and white bands. The estimated gas concentration changes much more quickly than the MOX conductance, thanks to the dynamic modeling and the phase switch detection performed by the aSLDS inference. During periods of exposure to gas, e.g., around 200 *s*, the estimated gas concentration varies according to the sensor trend, so small periods of *rise* and *recovery* are identified due to the turbulence in the environment. These are justified observing the PID measurements oscillations, which we consider being the ground truth. In periods where the signal is constant, e.g., between 300 *s* and 350 *s*, the switch probability graph show a gray band. This is explained by the fact that in absence of dynamical content of the signal, both phases are considered equally likely, which allows faster reaction in the estimation when the next transient occurs. A sensitive discrepancy between the estimated gas concentration an the PID measurement can be noted at the end of the recovery phases in all the experiments. This is probably due to an accumulation of gas in the experiment room, which affects the PID and the MOX sensors differently, due to the pre-processing and the different dynamic range of the sensors.

To evaluate quantitatively the performance of our gas concentration estimation approach, we compute the Normalized Root Mean Square Error (NRMSE) [36] between the estimated gas concentration time-series and the ground truth PID measurements for each sensor and for each validation experiment. To have an overall assessment of the performances, we average the NRMSE over all the sensors of the array. In order to have a term of comparison we also compute the NRMSE between the MOX measurements and the ground truth PID. Due to the slow dynamic response of MOX sensors, we expect this error to be higher than the one estimated using the aSLDS, especially for datasets with frequent changes in gas concentration. The direct comparison between the MOX measurement and the estimated/ground truth PID measurement requires a simple rescaling of the MOX measurements to the same range of the PID ones. Additionally, in order to evaluate the importance of the aSLDS transition model, where the switch probability is computed using the contributions of three terms (*i.e.*, the estimated trend *ẋ^s^*, the likelihood of the measurements given the two possible sensor phases and the prior probability of switch), we also compare our results to a *hard-switching* model. This model is somewhat similar to the one proposed in [12] because we assign values 1 and 0 to the switch variable depending on the sign of the estimated trend transforming the aSLDS treatment of the switching behavior from probabilistic to deterministic. The model is obtained simply modifying the original aSLDS transition model introduced in Section 4. In particular, we modify *p*(*s_t_*|*h_t_*_−1_, s*_t_*_−1_) , *i.e.*, the conditional distribution of the switch variable *s_t_*, as follows:

(19) $$\begin{array}{cc} \text{if}\;{\dot{x}}_{t-1}^{\text{s}}>0 & p({\text{s}}_{t}|{\text{s}}_{t-1},h_{t-1})= \end{array}\{\begin{array}{ll} 1 & \text{if}\;{\text{s}}_{t}=\text{rise}\wedge s_{t-1}=\text{rise}\\ 1 & \text{if}\;s_{t}=\text{rise}\wedge s_{t-1}=\text{recovery}\\ 0 & \text{if}\;s_{t}=\text{recovery}\wedge s_{t-1}=\text{rise}\\ 0 & \text{if}\;s_{t}=\text{recovery}\wedge s_{t-1}=\text{recovery} \end{array}$$

(20) $$\begin{array}{cc} \text{if}\;{\dot{x}}_{t-1}^{\text{s}}<0 & p({\text{s}}_{t}|{\text{s}}_{t-1},h_{t-1})= \end{array}\{\begin{array}{ll} 0 & \text{if}\;{\text{s}}_{t}=\text{rise}\wedge s_{t-1}=\text{rise}\\ 0 & \text{if}\;s_{t}=\text{rise}\wedge s_{t-1}=\text{recovery}\\ 1 & \text{if}\;s_{t}=\text{recovery}\wedge s_{t-1}=\text{rise}\\ 1 & \text{if}\;s_{t}=\text{recovery}\wedge s_{t-1}=\text{recovery} \end{array}$$

This model allows for faster switches, but as it will be shown, it is more sensitive to measurement noise and does not perform better than the fully probabilistic aSLDS in general.

**Figure 14.**
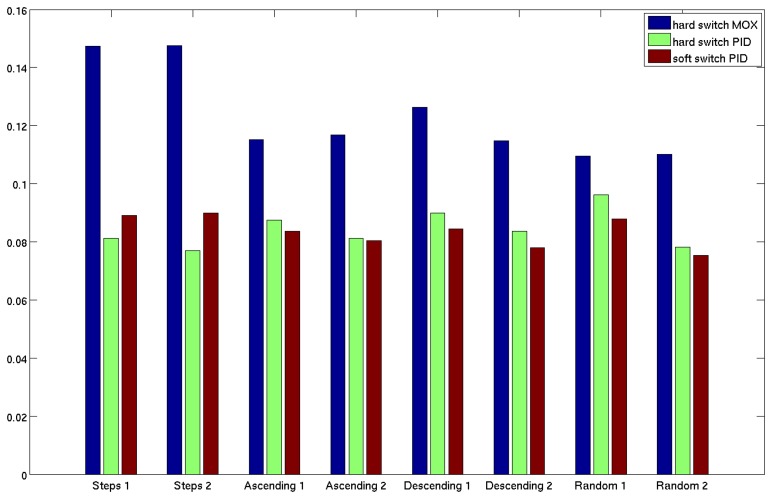
Normalized Root Mean Square error for each experiment, averaged over all sensors.

The bar graph in Figure 14 display the average NRMS error for each validation experiment, for the three aforementioned models. The re-scaled MOX measurements, as expected, lead to the biggest NRMS error. The hard switching model achieves a smaller error for the datasets *Steps*, because a faster switching model performs better than the soft switching one in correspondence of big jumps in gas concentration (like shown in Figure 9) but is, in general, penalized in the other cases, because the estimated derivative *ẋ^s^* (which is, in this model, the only responsible for driving switches in the sensor phase) is more sensitive to oscillations and noise in the MOX measurements.

## 6. Conclusions

In this paper, we applied a Bayesian time series model called the Augmented Switching Linear Dynamical System to the problem of estimating gas concentration in an Open Sampling System using MOX sensor. The proposed method is effective in overcoming one the biggest limitations of MOX sensors, namely their slow time-response, combining a system-modeling approach to probabilistic inference. We extend previous work in this field using the aSLDS to directly estimate the gas concentration from the MOX sensor, obtaining performances comparable to the ones obtainable using a more expensive and selective Photo Ionization Detector sensor.

The proposed aSLDS approach allows to model all the uncertainty involved in the experiment in a fully probabilistic framework, and to coherently include prior domain knowledge to simplify the problem. The method allows for future extension in multiple directions. For what concerns the model learning step, more complex dynamical model (*i.e.*, not limiting to first-order LDS) could be investigated, including for example, estimation of sensor drift. The tools to estimate the dynamical parameters in a fully Bayesian way are, to the best of the authors' knowledge, not yet available, at least in the case where a strong prior knowledge is available and we want the learned parameter values to be constrained by it. Nevertheless, this is indeed a hot topic in the Machine Learning community, so progress is to be expected.

As for the on-line inference, the aSLDS model is suitable to include extra variables into the estimation process, as long as their influence can be expressed in terms of conditional probability functions. For example, it could be possible to include include air humidity or temperature in the state vector, and model their influence on the sensor dynamical behavior. A less restraining assumption on the gas dynamic model could also be considered, for example moving from a constant model to constant-derivative models could better model the gas concentration oscillations due to turbulence in the flow.

To conclude, Bayesian time-series models are a natural choice where prior knowledge about the problem is available and can be quantified. For this reason, we believe that gas sensing represents an ideal application, and this synergy deserves further research.

## Figures and Tables

**Table 1.** Explanation of the proposed aSLDS transition model.

*p*(*s_t_*|*s_t_*_&minus 1_,*x_t_*_&minus 1_)	*s_t_* = *s_t_*_&minus 1_	*s_t_* ≠ *s_t_*_&minus 1_	Explanation
$(s_{t}=\text{rise}\wedge {\dot{x}}_{t-1}^{s}>0)\vee$ $\left(s_{t}=\text{recovery}\wedge {\dot{x}}_{t-1}^{s}\leq 0\right)$	*p*	1 &minus *p*	The estimated trend confirms the current phase, therefore favor self-transition
$(s_{t}=\text{rise}\wedge {\dot{x}}_{t-1}^{s}\leq 0)\vee$ $\left(s_{t}=\text{recovery}\wedge {\dot{x}}_{t-1}^{s}>0\right)$	*p* &minus *b*	1 − *p* + *b*	The estimated trend contradicts the current phase, therefore favor a phase switch
